# Early-life viral infection and allergen exposure interact to induce an asthmatic phenotype in mice

**DOI:** 10.1186/1465-9921-11-14

**Published:** 2010-02-03

**Authors:** Jessica S Siegle, Nicole Hansbro, Cristan Herbert, Helene F Rosenberg, Joseph B Domachowske, Kelly L Asquith, Paul S Foster, Rakesh K Kumar

**Affiliations:** 1 Department of Pathology, University of New South Wales, Sydney NSW 2052, Australia; 2 Centre for Asthma and Respiratory Disease, School of Biomedical Sciences, University of Newcastle, NSW 2300, Australia; 3 Hunter Medical Research Institute, Newcastle, NSW 2300, Australia; 4 Laboratory of Allergic Diseases, National Institute of Allergy and Infectious Diseases, National Institutes of Health, Bethesda MD 20892, USA; 5 Division of Infectious Diseases, Department of Pediatrics, SUNY Upstate Medical University, Syracuse, NY 13210, USA

## Abstract

**Background:**

Early-life respiratory viral infections, notably with respiratory syncytial virus (RSV), increase the risk of subsequent development of childhood asthma. The purpose of this study was to assess whether early-life infection with a species-specific model of RSV and subsequent allergen exposure predisposed to the development of features of asthma.

**Methods:**

We employed a unique combination of animal models in which BALB/c mice were neonatally infected with pneumonia virus of mice (PVM, which replicates severe RSV disease in human infants) and following recovery, were intranasally sensitised with ovalbumin. Animals received low-level challenge with aerosolised antigen for 4 weeks to elicit changes of chronic asthma, followed by a single moderate-level challenge to induce an exacerbation of inflammation. We then assessed airway inflammation, epithelial changes characteristic of remodelling, airway hyperresponsiveness (AHR) and host immunological responses.

**Results:**

Allergic airway inflammation, including recruitment of eosinophils, was prominent only in animals that had recovered from neonatal infection with PVM and then been sensitised and chronically challenged with antigen. Furthermore, only these mice exhibited an augmented Th2-biased immune response, including elevated serum levels of anti-ovalbumin IgE and IgG_1 _as well as increased relative expression of Th2-associated cytokines IL-4, IL-5 and IL-13. By comparison, development of AHR and mucous cell change were associated with recovery from PVM infection, regardless of subsequent allergen challenge. Increased expression of IL-25, which could contribute to induction of a Th2 response, was demonstrable in the lung following PVM infection. Signalling via the IL-4 receptor α chain was crucial to the development of allergic inflammation, mucous cell change and AHR, because all of these were absent in receptor-deficient mice. In contrast, changes of remodelling were evident in mice that received chronic allergen challenge, regardless of neonatal PVM infection, and were not dependent on signalling via the IL-4 receptor.

**Conclusion:**

In this mouse model, interaction between early-life viral infection and allergen sensitisation/challenge is essential for development of the characteristic features of childhood asthma, including allergic inflammation and a Th2-biased immune response.

## Background

Asthma is one of the most common chronic diseases in children in industrialised societies [1]. The association between childhood infections and asthma is complex, because at least in some settings, repeated exposure to infectious agents may reduce the likelihood of developing allergic diseases [2]. However, epidemiological studies strongly suggest that lower respiratory viral infections associated with wheezing, especially in early childhood, play an important role in the subsequent development of asthma in children who are repeatedly exposed to inhaled allergens [3-8].

Respiratory syncytial virus (RSV) infection, particularly severe disease with bronchiolitis in children under 1 year, has a strong linkage to allergic asthma [9,10]. The underlying mechanisms remain unknown, in large part because investigation in animal models is confounded by the species specificity of pneumoviruses. There is little if any replication of human RSV in mice, so that murine models using this virus require a very large inoculum (>10^4 ^pfu) [11,12]. By comparison, infection with pneumonia virus of mice (PVM), which belongs to the same family (*Paramyxoviridae*) and genus (*Pneumovirus*) as RSV [13], provides a realistic model of RSV disease. Because PVM is a natural rodent pathogen, far fewer virions are required to induce infection. As little as 2 pfu elicits a respiratory tract infection, ~30 pfu provokes a productive infection with severe bronchiolitis, and 300 pfu results in rapid death [11]. In its full-blown form, PVM infection induces severe pulmonary inflammation with recruitment of neutrophils and marked oedema, thus resembling acute respiratory distress syndrome [14]. This is paralleled by marked respiratory dysfunction as assessed by whole body plethysmography, which is accompanied by local production of pro-inflammatory mediators including MIP-1α, MIP-2, MCP-1 and IFN-γ [15]. MIP-1α and IFN-γ are crucial to granulocyte recruitment [14,16].

Investigating the link between an early-life viral infection and childhood asthma also requires an appropriate model of allergen sensitisation and long-term challenge. We have previously established murine models of mild chronic asthma and an allergen-induced acute exacerbation [17,18]. Hallmarks of these models include control of the mass concentration of aerosolised antigen to which animals are exposed, facilitating induction of lesions corresponding in their spatial distribution to those observed in the human disease. Low-level challenge over periods of 4 or more weeks induces chronic inflammation and remodelling which in contrast to other models is limited to conducting airways, with minimal parenchymal inflammation. Subsequent delivery of a single moderate-level challenge elicits features of an acute exacerbation, characterised by distal airway inflammation with eosinophil recruitment [18].

In this study, we have employed a unique combination of these models to provide direct experimental evidence for an interaction between early-life infection with a pneumovirus and allergen sensitisation/challenge in the development of an asthmatic phenotype. We found that key features of the asthmatic response, notably eosinophil recruitment and an associated Th2 cytokine response, were detected only in the groups of mice that had recovered from neonatal infection with PVM and were then subjected to chronic antigen challenge. Induction of these features was abrogated in mice deficient in their ability to express the IL-4 receptor α chain, indicating a crucial role for signalling via this receptor. Furthermore, we found that IL-25 was upregulated following viral infection, suggesting a possible mechanism by which a Th2-biased response might be triggered. This novel experimental system provides opportunities for investigation of pathogenetic mechanisms of childhood asthma, as well as for potential interventions.

## Methods

### Animals

Pregnant specific pathogen-free wild-type BALB/c mice were obtained from Animal Services, University of Newcastle. To assess the contribution of signalling via the IL-4 receptor, which is utilised by both IL-4 and IL-13, responses were compared in mice on the same genetic background but deficient in their ability to express the IL-4 receptor α chain (IL-4Rα^-/-^) [19]. During infection and allergen sensitisation, neonatal and weanling mice were maintained in individually ventilated cages in the animal housing facility, David Maddison Building, University of Newcastle. At 5-6 weeks of age, mice were transported to the University of New South Wales where they were maintained in a laminar flow holding unit (Gelman Sciences, Australia) and housed in autoclaved cages, in an air-conditioned room on a 12 hour light/dark cycle. Additional control animals, specific pathogen-free female BALB/c mice aged 8 weeks, were purchased from the Animal Resources Centre, Perth and held at the University of New South Wales throughout the duration of the study. Irradiated food and acidified water were provided *ad libitum *throughout. All experimental procedures complied with the requirements of the Animal Care and Ethics Committee of the University of New South Wales (ref no. 09/116B) and the University of Newcastle (ref no. 1026).

### PVM infection, allergen sensitisation and inhalational challenge

**Figure 1:**
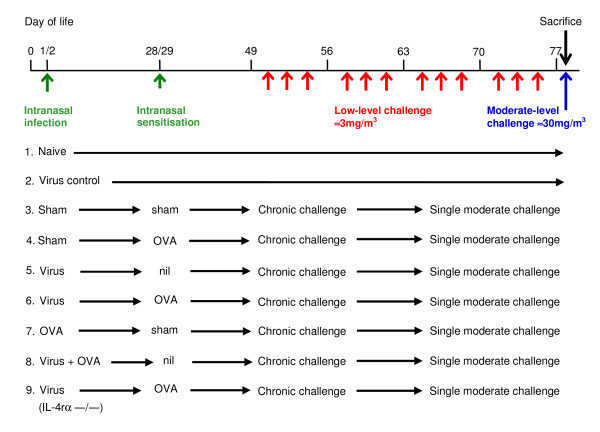
**Experimental design**. Diagrammatic representation of protocol for infection, sensitisation and inhalational challenge.

The various experimental and control groups used in preliminary studies to establish the model are shown in Figure 1. These permitted assessment of the effects of recovery from neonatal viral infection, sensitisation at different time points, and chronic inhalational challenge, either alone or in combination. For each experiment, groups comprised 8 mice, and all findings were confirmed in at least 2 separate experiments. Infection of neonatal mice with PVM used mouse passaged PVM J3666 strain (~1 × 10^5 ^pfu/ml). On both days 1 and 2 of life, mice were intranasally inoculated with 2 pfu in 5 μl phosphate buffered saline (PBS) on the external nares. Control animals were sham-infected with PBS alone. Intranasal sensitisation to chicken egg ovalbumin (Grade V, ≥ 98% pure, Sigma Australia, Australia) was performed either at days 1 and 2 of life or at days 28 and 29, with 5 μg OVA/5 μl PBS or 100 μg/40 μl respectively (Figure 1).

Inhalational challenge to aerosolised ovalbumin was performed as previously described [17,18]. Briefly, commencing at 7 weeks of age, BALB/c mice or IL-4Rα^-/- ^mice received low-level aerosol challenge with ovalbumin (mass concentration of ≈ 3 mg/m^3 ^of ovalbumin for 30 min/day, 3 days/week for 4 weeks). This was followed by a single moderate-level challenge (≈ 30 mg/m^3 ^for 30 minutes) to induce the changes of an acute exacerbation (Figure 1). During inhalation exposures, mice were held in flow-through wire cage racks (Unifab Corporation, Kalamazoo, Michigan, USA). Filtered air was drawn through the inhalation chamber (0.5 m^3^) at a flow rate of 250 L/min, and an aerosol of ovalbumin was generated from a 2.5% solution by controlled delivery of compressed air to a sidestream nebuliser (Trimed, Australia). Particle concentration within the chamber was continuously monitored using a DustTrak 8520 instrument (TSI, St Paul, MN).

### Bronchoalveolar lavage and histopathology

Four hours after the final aerosol challenge, mice were killed by exsanguination following an overdose of sodium pentobarbital. Bronchoalveolar lavage (BAL) fluid was collected and total cell counts were performed. Differential cell counts were assessed on at least 300 cells in a Leishman-stained smear [18].

Following perfusion and BAL fluid collection, the left lung was inflated and fixed in 10% buffered formalin [17]. The longitudinally orientated trachea and a horizontal slice from the mid zone were embedded in paraffin and used to assess inflammation and changes of remodelling. The right lung was collected, diced into small fragments and stored in Tri-reagent for RNA extraction. An additional 8 animals per group had 0.8 - 1.0 ml OCT compound (ProSciTech, Australia) instilled into the left lung, with a horizontal slice from the mid zone collected and frozen in liquid nitrogen.

### Morphometry

Immunoperoxidase staining for CD3^+ ^T-lymphocytes in frozen lung tissue was performed using rabbit anti-human antibodies against CD3 (Sigma-Aldrich, Australia). Eosinophils in lung tissue were identified using a cyanide-resistant peroxidase staining technique [20,21]. Briefly, acetone fixed, frozen lung sections were incubated in a 5 mM potassium cyanide solution for 10 minutes to inhibit the myeloperoxidase activity of neutrophils and macrophages. Following a thorough wash, slides were incubated in diaminobenzidine solution for 5 minutes and counterstained with methyl green. Eosinophils were quantified at 40× magnification using Spot imaging software. Results were expressed as number of cells per mm^2 ^of parenchymal tissue.

Inflammation in the airway wall was quantified in haematoxylin and eosin-stained sections of trachea. Intraepithelial eosinophils and nuclear profiles in the lamina propria were counted and the length of the epithelial basement membrane was measured at 40× magnification. Results were expressed as number of cells per 100 μm basement membrane, as previously described [17]. Lymphoid aggregates adjacent to small airways were enumerated in haematoxylin and eosin-stained sections of lung tissue.

Subepithelial collagenisation and epithelial hypertrophy were assessed in reticulin stained trachea as previously described [17]. Goblet cell changes were assessed in periodic acid schiff (PAS) stained lung tissue. The largest visible airway, usually the left main bronchus, was assessed in each section. Positively stained cells were semi-quantitatively graded on a scale from 0-4, where a grade of 0 = <1% positive cells, 1 = 1-3%, 2 = 4-10%, 3 = 11-30%, and 4 = ≥ 31%.

### Airway hyperresponsiveness

Airway responsiveness to inhaled β-methacholine was measured by assessing transpulmonary resistance (R_L_) 4 hours after the final aerosol challenge [18,22]. Mice were challenged with saline to establish baseline responses, then with increasing concentrations of β-methacholine (6.25, 12.5, 25 and 50 mg/ml). Aerosols were generated with an ultrasonic nebuliser (Buxco, Aeroneb Laboratory Nebulizer) and delivered to the inspiratory line. Each aerosol was delivered for a period of 5 minutes and a computer program (BioSystemXA, Buxco Electronics, Inc.) was used to calculate pulmonary resistance from the continuously recorded pressure and flow data. Peak pulmonary resistance values were taken as the maximum response to the concentration of methacholine being tested, and are expressed as the percent change over the saline control. Dose-response curves were plotted for each animal and the area under the curve was calculated.

### Cell isolation

CD4^+ ^T-lymphocytes were isolated from pooled pairs of lungs four hours after the final challenge. Briefly, lung tissue was diced into fine fragments (<0.3 mm^3^) and dispersed in a mixture of Type IV collagenase (Worthington Biochemical, Lakewood, NJ) and DNase (Roche Diagnostics, Australia). Following incubation on a roller mixer for 40 minutes at 37°C, samples were vortexed and passed through a 70 μm cell strainer. Cells were re-suspended in red cell lysis buffer and washed with PBS. This yielded a mixed population of cells, the majority of which were lymphocytes and macrophages. Cell viability was >90% in all samples. CD4^+ ^T cells were then isolated using a Mouse CD4 FlowComp™ magnetic bead isolation kit (Invitrogen, Carlsbad, CA) according to the manufacturer's instructions. This method yields ~98.0% pure CD4^+ ^T cell populations. A total of 5 × 10^5 ^cells were suspended and lysed in Tri-reagent (Sigma Australia, Australia) for RNA extraction, while the remaining cells were kept on ice until ready to use in enzyme-linked immunospot (ELISpot) assays. From some groups of animals, blood was also collected by cardiac puncture and serum was stored at -20°C for later analysis.

### Enzyme immunoassays

PVM-specific IgG antibodies were measured from sham-infected and virus-infected animals using the SMART M12 kit (Biotech Trading Partners, El Cerrito, CA). Briefly, appropriate controls and serum samples were incubated at room temperature for 30 minutes. Wells were washed 5 times after this and each subsequent incubation. One drop of enzyme conjugate was added to each well and incubated for 30 minutes, which was followed by the chromogen substrate for 10 minutes. Reactions were subsequently stopped with phosphoric acid, and plates were read on a Bio-Rad microplate reader (680XR) at 450 nm.

OVA-specific IgE in serum was measured using a commercial kit (MD Biosciences, St Paul, MN) according to the manufacturer's instructions. Ovalbumin-specific IgG_1 _in serum was measured as previously described [23]. Briefly, appropriate wells were coated with either anti-IgG_1 _(BD Pharmingen) or ovalbumin (2 μg/well). After blocking, wells were incubated with serial dilutions of serum or standards (mouse IgG_1_), followed by detection with streptavidin HRP anti-IgG_1_. Plates were developed and read at 450 nm using a BioRad 680 Microplate reader. Samples were measured in duplicate and the sensitivity of detection was 3 ng/ml.

### Enzyme-linked immunospot assays

Cytokine secretion by CD4^+ ^T cells was assessed using MultiScreen_HTS_-HA Filter Plates (Millipore, Australia) coated with appropriate antibodies (R&D Systems, Australia) according to the manufacturer's instructions. Isolated CD4^+ ^T cells were cultured in RPMI 1640 (containing 10% FBS, 1% penicillin/streptomycin, 1% L-glutamine, 1% Non-essential amino acids, 1% sodium pyruvate) at 2 × 10^5 ^cells/well in the presence of 1 mg/ml ovalbumin for 24 hours. Appropriate detection antibodies (R&D Systems, Australia) were added to wells and the plate was incubated overnight. Following washing, a working solution of streptavidin-AP (1:1000) (MabTech Australia Pty Ltd, Australia) was added to each well and left at room temperature for 2 hours. Subsequently, BCIP/NBT (Sigma Australia, Australia) was used to develop spots, which took up to 30 minutes. Spots were detected using the AID ELISpot reader (Autoimmun Diagnostika, Germany).

### RNA isolation and polymerase chain reaction

Extraction of mRNA from whole lung and CD4^+ ^T cells was performed using Tri-reagent (Sigma Australia, Australia) according to the manufacturer's instructions. RNA concentrations were measured using a NanoDrop ND-100 spectrophotometer (NanoDrop Technologies, Wilmington, DE). Viral load was assessed 2, 4, 7, 14, 21 and 28 days post infection in whole lung. Samples were reverse transcribed with the Transcriptor First Strand cDNA Synthesis Kit (Roche Diagnostics, Australia) and M-MLV-RT (Invitrogen). Expression of PVM was assessed by real time PCR, with SYBR green to detect amplified products. Primer sequences were as follows: PVM forward 5'-GCCTGCATCAACACAGTGTGT-3' and reverse 5'-GCCTGATGTGGCAGTGCTT-3' [24,25]. IL-25 levels were similarly assessed in whole lung at 14 and 28 days, while *Gob5 *levels were assessed in whole lung at day 78.

For isolated CD4^+ ^T cells, mRNA was treated with Turbo DNase (Ambion Inc., Austin, TX, USA) and samples reverse transcribed using SuperScript^® ^III First Strand Synthesis System (Invitrogen, Australia) according to the manufacturer's instructions. Expression of cytokines and transcription factors was assessed via quantitative RT-PCR, with SYBR green used to detect amplified products. Primers were either kindly provided by Dr. Ken Hsu (University of New South Wales), taken from an external reference [26] or designed using Primer Express software (Applied Biosystems, Australia). Real-time reactions were performed using an ABI Prism 7700 Sequence Detection System (Applied Biosystems, Australia), and gene expression was normalised to hypoxanthine phosphoribosyltransferase (HPRT).

### Statistical Analysis

Results are presented as arithmetic mean (S.E.M.) or as medians (interquartile range). For comparison between multiple groups, differences compared to the relevant control group were assessed using one-way ANOVA or the corresponding non-parametric Kruskal-Wallis test, followed by either a Dunnett's test or Bonferroni-corrected t tests as appropriate. The software package GraphPad Prism (GraphPad Software, San Diego, California, USA) was used for all data analysis and preparation of graphs.

## Results

### PVM infection and recovery

**Figure 2:**
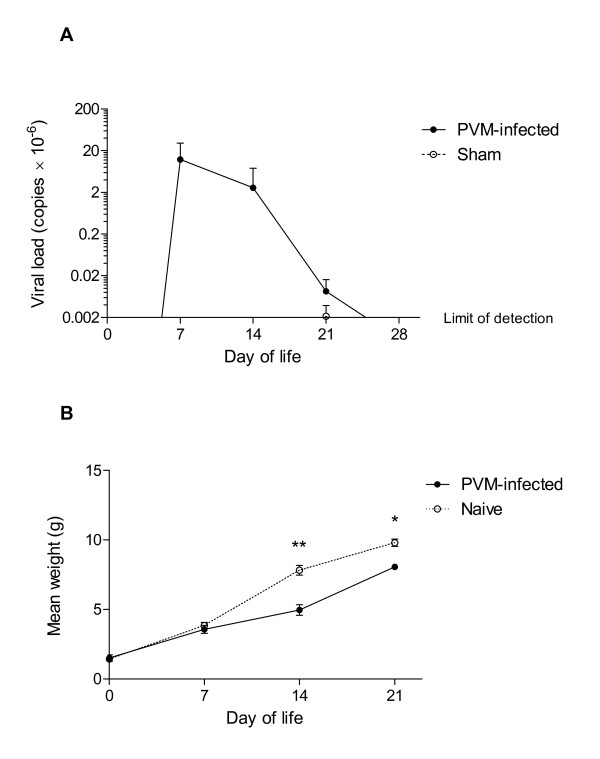
**PVM infection**. *(A) *PVM load assessed by PCR of lung tissue at 2-28 days post-infection. Levels of virus in samples from infected animals at days 2, 4 and 28 were at or below the limits of detection, as were levels in all samples from sham-infected control animals. *(B) *Mean weights of PVM-infected and naïve mice during the first 3 weeks of life. Significant differences shown as * = P < 0.05, ** = P < 0.01.

Intranasal inoculation of PVM at days 1 and 2 of life effectively established viral infection, with viral load peaking at day 7 (Figure 2A). By day 14 viral load had decreased substantially, with clearance achieved at 4 weeks after infection. The mice did not develop any visible clinical manifestations of infection and there was no excess mortality. However, mean weight was significantly decreased at 14 and 21 days after infection (Figure 2B). Enumeration of BAL cells in 14 day old mice revealed an increased total number of cells, as well as increases in the numbers of neutrophils, eosinophils and lymphocytes (not shown). Viral infection led to seroconversion, assessed at 11 weeks following infection (not shown).

### Development of lesions in response to infection and/or allergen exposure

#### Inflammatory response

A modest increase in the number of CD3^+ ^T cells in lung tissue, identified by immunostaining, was noted following either an early-life viral infection alone or ovalbumin sensitisation and challenge, although these increases were not statistically significant (Table 1). In contrast, the combination of recovery from PVM infection and OVA challenge, with or without intranasal sensitisation at days 28 and 29, led to a significant increase in the number of intrapulmonary T cells compared to naïve animals (P < 0.05). By comparison, OVA sensitisation at days 1 and 2, with or without viral infection, was not associated with a significant increase in CD3^+ ^T cells in response to chronic challenge (Table 1).

**Table 1:** Inflammatory and epithelial changes in the airways and lungs

	Experimental groups								
	Naive	Virus control	Sham/sham	Sham/OVA	Virus/nil	Virus/OVA	OVA/sham	Virus+OVA/nil	Virus/OVA IL-4Rα^-/-^
Intrapulmonary CD3^+ ^T cells/mm^2^	2.8 (0.8)	7.2 (0.9)	3.3 (0.6)^#^	8.0 (1.3)	10.4 (3.2) *	10.2 (2.2) *	4.4 (0.9)	6.6 (0.8)	4.7 (1.2)
Intrapulmonary eosinophils/mm^2^	2.7 (0.6)	3.7 (0.9)^#^	1.5 (0.4)^###^	3.7 (1.1)^#^	3.0 (0.8)^#^	6.5 (0.6) ***	1.1 (0.5)^###^	5.7 (0.9)	0.9 (0.3)^###^
Lymphoid aggregates per section^§^	0.0 (0.75)	2.0 (3.25) *	0.0 (0.25)	0.0 (1.25)	1.0 (4.0)	1.0 (2.0)	0.5 (1.0)	1.0 (1.25)	2.5 (4.75) **
Lamina propria cells/100 μm basement membrane	19.2 (1.0)	22.3 (1.1)	19.6 (1.7)^#^	24.0 (1.2) *	24.8 (1.5) *	25.1 (1.2) *	20.0 (1.5)	22.0 (1.6)	21.7 (1.0)
Thickness of subepithelial collagen zone (μm)	3.2 (0.2)	4.0 (0.2)	2.7 (0.2)^###^	4.7 (0.2) ***	4.3 (0.2) **	4.8 (0.1) ***	4.2 (0.2) *	4.2 (0.2) *	4.4 (0.4) **
Thickness of epithelial cell layer (μm)	12.5 (0.5)	12.6 (0.8)^#^	12.1 (0.7)^##^	16.9 (0.5) ***	15.1 (0.7)	16.1 (1.3) *	14.6 (0.7)	13.7 (0.9)	16.2 (0.6) *
Grade of mucous cell metaplasia^§^	1.0 (1.0)	3.0 (3.0)*	0.0 (1.0)^##^	2.0 (2.0)	3.0 (2.0) **	3.0 (1.0) **	2.0 (2.0)	1.0 (2.0)	0.0 (0.0)^###^

Data are presented as mean (S.E.M), except ^§ ^which are median (interquartile range). Significant differences compared to naïve animals shown as * = P < 0.05, ** = P < 0.01, *** = P < 0.001; compared to virus-infected, OVA-challenged animals (Virus/OVA) shown as ^# ^= P < 0.05, ^## ^= P < 0.01, ^### ^= P < 0.001.

**Figure 3:**
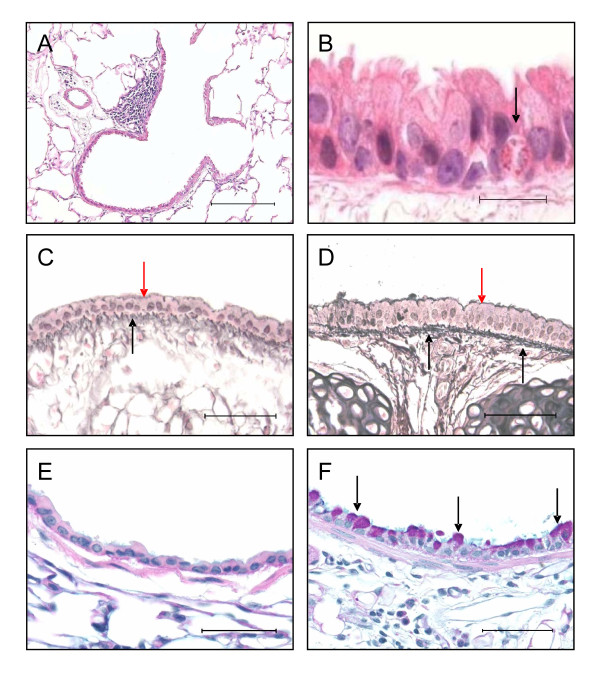
**Histopathological changes**. *(A) *A lymphoid aggregate adjacent to a bronchiole, in lung tissue from an animal from the virus-infected control group at 11 weeks of age. *(B) *An eosinophil within the tracheal epithelium (arrow) from a mouse from the virus/OVA group. *(C) *Reticulin-stained trachea from a naïve animal demonstrating normal thickness of subepithelial collagenous zone (black arrow) and epithelial layer (red arrow). *(D) *Trachea from a mouse infected with PVM at birth, then sensitised to OVA and challenged (virus/OVA group) demonstrating significant thickening of both the subepithelial collagenous zone (black arrows) and the epithelium (red arrow). *(E) *Left main bronchus from a naïve animal stained with PAS, showing that no mucin-producing cells are present (grade 0). *(F) *Left main bronchus from a mouse from the virus/OVA group, demonstrating numerous PAS-positive goblet cells in the epithelium (arrows) (grade 4). *Scale bar *= 150 μm in A, 25 μm in B and 50 μm in C-F.

Recovery from PVM infection was associated with the presence of small lymphoid aggregates in the lung tissue, usually adjacent to small airways, with the typical morphology of inducible bronchus-associated lymphoid tissue (iBALT) including a peripheral zone of CD3-positive cells (Figure 3A) [27]. Numbers of iBALT aggregates were somewhat reduced in mice that were sensitised and challenged with OVA, except in the case of IL-4Rα^-/- ^mice (Table 1).

Acute allergic inflammation, with accumulation of eosinophils within airway epithelium (Figure 3B) and in lung tissue, was demonstrable in animals that recovered from an early-life PVM infection and then received OVA sensitisation at days 28 and 29 followed by chronic challenge. Numbers of intrapulmonary eosinophils were significantly increased only in the virus/OVA group (P < 0.01) (Table 1).

In the lamina propria of the conducting airways, significantly increased numbers of chronic inflammatory cells were evident in chronically challenged mice following either recovery from PVM infection, sensitisation with OVA at days 28 and 29, or both (P < 0.05). In animals which were sensitised at days 1 and 2 of life, there was no such increase in the number of inflammatory cells (Table 1).

The role of signalling via the IL-4 receptor was demonstrated by the observation that in IL-4Rα^-/- ^mice which recovered from an early-life PVM infection and then received OVA sensitisation followed by chronic challenge, development of both acute and chronic components of the allergic inflammatory response was completely suppressed (Table 1).

#### Airway wall remodelling

Subepithelial collagenisation (at least P < 0.01) was apparent in all groups of mice that were chronically challenged with OVA, except the group of sham infected and sensitised animals (Table 1 and Figure 3C, D). There were no significant changes in mice that recovered from neonatal PVM infection but were not sensitised or challenged. Significant epithelial hypertrophy (at least P < 0.05) was evident in mice sensitised with OVA at days 28 and 29 of life and chronically challenged (Table 1 and Figure 3C, D), but not in those sensitised at days 1 and 2 of life. Again, no significant changes were observed in mice that recovered from neonatal PVM infection but were not sensitised or challenged. Changes of remodelling were not related to signalling via the IL-4 receptor, as responses similar to those seen in the virus/OVA group were also observed in the infected, sensitised and challenged IL-4Rα^-/- ^mice (Table 1).

**Figure 4:**
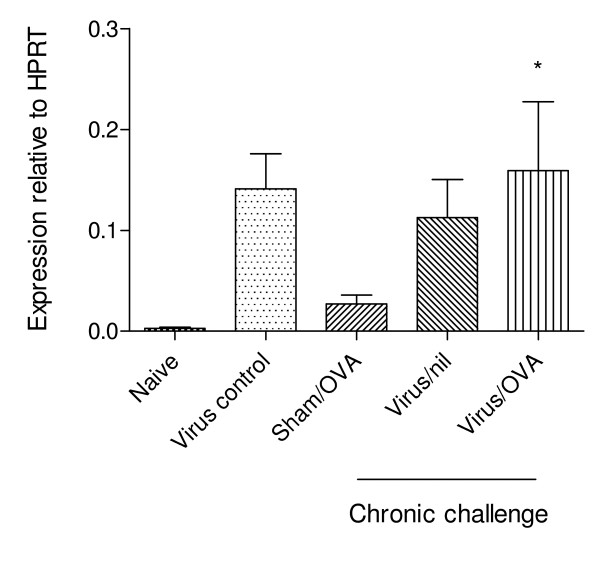
**Expression of *Gob5***. mRNA expression for *Gob5 *assessed by PCR of lung tissue. Significant difference compared to naïve animals shown as * = P < 0.05.

Mucin-secreting PAS positive cells were infrequently observed in the epithelium of naïve mice (Figure 3E). Significant metaplasia/hyperplasia of goblet cells (at least P < 0.05) was associated with PVM infection at birth (Table 1 and Figure 3F), while a lesser increase (not statistically significant) was also observed in OVA-sensitised and challenged animals that had not had early-life infection with PVM. Corresponding with mucous cell changes, expression of mRNA for *Gob5 *was significantly increased (approximately 50 fold, P < 0.05 for the virus/OVA group) in the lung tissue of animals that had recovered from viral infection (Figure 4). Mucous cell metaplasia/hyperplasia was much less marked in animals sensitised at days 1 and 2 of life and was completely absent in the infected, sensitised and challenged IL-4Rα^-/- ^mice (Table 1).

#### Airway responsiveness

**Figure 5:**
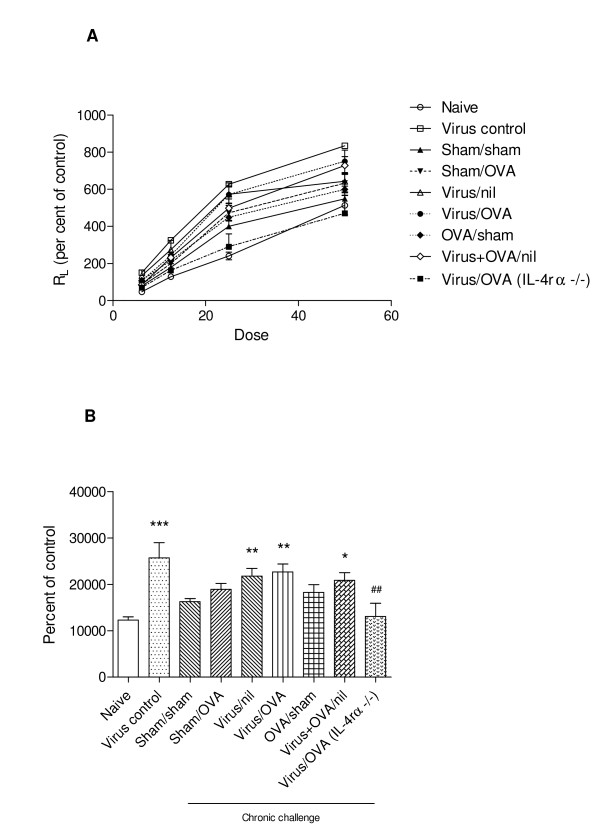
**Development of AHR**. *(A) *Airway responsiveness to increasing concentrations of β-methacholine, assessed by transpulmonary resistance (R_L_). *(B) *Transpulmonary resistance assessed as area under the curve. Significant differences compared to naïve animals shown as * = P < 0.05, ** = P < 0.01, *** = P < 0.001; compared to virus-infected, OVA-challenged animals (Virus/OVA) shown as ## = P < 0.01.

At 4 hours after the final challenge, all groups of wild type mice that had recovered from PVM infection exhibited a significant increase in R_L_, compared to naïve control animals (Figure 5). This was dependent on signalling via the IL-4 receptor, as AHR was absent in infected, sensitised and challenged IL-4Rα^-/- ^mice. Notably, OVA sensitisation and challenge without early-life viral infection did not elicit AHR.

### Interaction between infection and allergen exposure

To investigate the interaction between infection and chronic allergen challenge, airway and pulmonary inflammation were assessed in parallel with cytokine and transcription factor expression by pulmonary CD4^+ ^T cells. These studies excluded animals sensitised at days 1 and 2, which exhibited down-regulated responses. Because the responses of sham-infected and sham-sensitised animals were indistinguishable from those of naïve animals, the latter were used as controls for these experiments.

#### Inflammatory response

**Figure 6:**
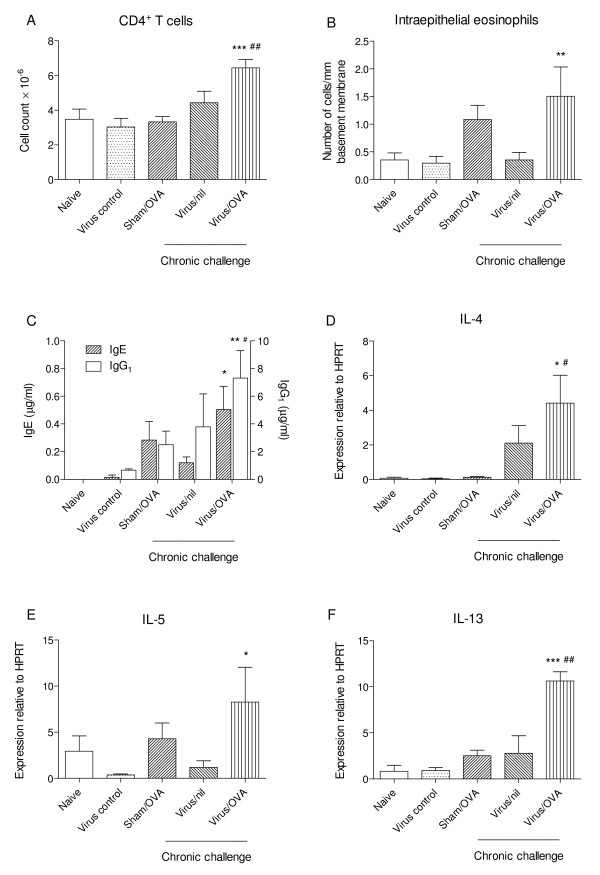
**Th2-biased immunological response**. *(A) *Numbers of CD4^+ ^T cells recovered from disaggregated lung tissue. *(B) *Numbers of eosinophils in tracheal epithelium. *(C) *Levels of OVA-specific IgE and IgG_1 _in serum. *(D-F) *Levels of expression of mRNA for IL-4, IL-5 and IL-13 by CD4^+ ^T-cells, relative to HPRT. Significant differences compared to virus-infected control animals shown as * = P < 0.05, ** = P < 0.01, *** = P < 0.001; compared to sham-infected, OVA-challenged animals (Sham/OVA) shown as # = P < 0.05, ## = P < 0.01.

Cells from BAL were collected for total and differential counts, while pulmonary CD4^+ ^cells recovered from disaggregated tissue and intraepithelial eosinophils in sections of trachea were enumerated in parallel. As shown in Table 2, a markedly increased yield of BAL cells was observed only in the virus/OVA chronically challenged group and this was significantly greater than in either the virus control group or the sham/OVA chronically challenged group (P < 0.001 for both comparisons). Paralleling these observations was a significant increase in the recovery of CD4^+ ^cells from the virus/OVA group (P < 0.001 compared to the virus control group, P < 0.01 compared to the sham/OVA group, Figure 6A). Similarly, as shown in Figure 6B, numbers of intraepithelial eosinophils were highest in the virus/OVA group (P < 0.01 compared to the virus control group) although a moderate increase was also noted in the sham/OVA group.

**Table 2:** BAL cellular response

Groups	Total	Macrophages	Lymphocytes	Neutrophils	Eosinophils
Naïve	263 (34.1)	259 (1.8)	3 (1.3)	0 (0.3)	1 (0.8)
		98.3% (0.7)	1.3% (0.5)	0.1% (0.1)	0.3% (0.3)
Virus control	312 (19.8)	262 (5.6)	47 (4.7)	1 (0.3)	1 (0.3)
		84.1% (1.8) ***	15.1% (1.5) ***	0.2% (0.1)	0.2% (0.1)
Sham/OVA	171 (20.5)	145 (4.8)	24 (3.4)	2 (1.7)	0 (0.2)
		84.7% (2.8) ***	14.0% (2.0) ***	1.2% (1.0)	0.1% (0.1)
Virus/nil	330 (41.7)	275 (5.3)	47 (3.3)	7 (3.3)	0 (0.3)
		83.3% (1.6) ***	14.3% (1.0) ***	2.2% (1.0)	0.1% (0.1)
Virus/OVA	555 (60.0) ***	429 (11.6)	104 (11.6)	14 (2.75)	2 (0.6)
		78.0% (2.1) ***	18.9% (2.1) ***	2.6% (0.5) *	0.3% (0.1)

Data are presented as mean numbers of cells × 10^-3 ^(S.E.M) and as percentages for individual cell types (n = 8). Significant differences compared to naïve animals shown as * = P < 0.05, ** = P < 0.01, *** = P < 0.001.

#### Anti-OVA IgE and IgG_1 _response

Serum levels of ovalbumin-specific IgE were significantly increased only in the virus/OVA chronically challenged group (P < 0.05 compared to the virus control group) (Figure 6C). Similarly, levels of ovalbumin-specific IgG_1_, which is induced in parallel with IgE in mice [28], were most strikingly increased in the virus/OVA chronically challenged group (P < 0.01 compared to the virus control group, P < 0.05 compared to the sham/OVA group) (Figure 6C).

#### Pulmonary CD4^+ ^T cell cytokine profile

Assessment of the expression of Th1 and Th2 cytokines and their corresponding transcription factors revealed that only in mice which had recovered from early-life PVM infection and then been sensitised and chronically challenged with OVA was there a Th2-biased immunological profile. In CD4^+ ^T cells from these animals, there was significantly elevated relative expression of IL-4 (P < 0.05 compared to both the virus control group and the sham/OVA group), IL-5 (P < 0.05 compared to the virus control group) and IL-13 (P < 0.001 compared to the virus control group, P < 0.01 compared to the sham/OVA group) (Figure 6D-F). In contrast, recovery from PVM infection or OVA sensitisation and challenge were not by themselves associated with significantly increased Th2 cytokine expression, compared to naïve animals. As expected, no increase in expression of Th2 cytokines was demonstrable in IL-4Rα^-/- ^mice (not shown).

Expression levels of GATA-3, the Th2 associated transcription factor, mimicked the pattern of the major Th2 cytokines, although the magnitude of these changes was not statistically significant. Levels of IFN-γ expression were not increased in any of the experimental groups, nor was there any increase in the expression of T-bet, the Th1 associated transcription factor (data not shown).

Because our models involve challenge with the relatively low mass concentrations of aerosolised OVA, assessment of levels of inflammatory cytokines in BAL fluid is not feasible. Therefore, we attempted to assess secretion of Th1 and Th2 cytokine proteins in supernatants of short-term cultures of CD4^+ ^T cells restimulated with OVA ex vivo, but these concentrations also fell below the limits of detection in a multiplex assay. Accordingly, we assessed secretion by isolated CD4^+ ^T cells using ELISpot assays. Only cells from animals that had recovered from PVM infection and then been sensitised and challenged with OVA exhibited significantly increased secretion of IL-4 and IL-13 (Table 3). These animals also exhibited enhanced secretion of IL-5, although this increase was not statistically significant. In contrast, there was no evidence of increased secretion of IFN-γ in any of the experimental groups.

**Table 3:** Cytokine secretion by CD4^+ ^T cells

	IL-4	IL-5	IL-13	IFN-γ
Naïve	6 (7.6)	28 (6.8)	9 (7.9)	28 (3.8)
Virus control	18 (6.3)	33 (3.8)	16 (5.8)	25 (4.6)
Sham/OVA	10 (5.2)	18 (4.5)	11 (3.7)	6 (2.2)
Virus/nil	26 (7.9)	28 (9.6)	31 (8.2)	31 (6.1)
Virus/OVA	43 (13.1) *	58 (18.0)	49 (7.4) **	15 (4.1)

Data are presented as mean numbers of spot-forming cells (S.E.M). Significant differences compared to naïve animals shown as * = P < 0.05, ** = P < 0.01.

#### Expression of IL-25 in lung tissue

To assess the possible role of epithelial injury by early-life PVM infection in the induction of a Th2-biased immunological response, we assessed relative expression of mRNA for IL-25 in lung tissue at days 14 and 28 of life. As shown in Table 4, IL-25 expression at day 14 was significantly up-regulated (P < 0.001 compared to sham-infected animals, *t*-test). Levels were also increased at day 28 but because of the scatter of individual sample values, this increase was not statistically significant.

**Table 4:** Relative expression of mRNA for IL-25 in lung tissue

Day	Sham control	PVM infected
14	1.13 (0.21)	4.05 (0.57) ***
28	144.1 (76.5)	1005.8 (841)

Data are presented as copies per 10^3 ^copies of HPRT (S.E.M). Significant difference shown as *** = P < 0.001.

## Discussion

Epidemiological studies have clearly demonstrated an association between early-life viral infections, allergen exposure and subsequent development of childhood asthma. While these factors appear to be synergistic [4], the relationship remains poorly understood [29] and is difficult to investigate in the absence of an appropriate experimental model. In this study we have, for the first time, demonstrated this interaction in a clinically relevant model of childhood asthma. Our model has particular advantages because it employs PVM, a species-specific pneumovirus that replicates in mouse lung tissue, induces marked inflammation and thus models the full spectrum of pathological changes of human RSV disease in early life [30,31]. This is in contrast to murine experimental models based on human RSV, which utilise extraordinarily high inocula but still achieve little or no virus replication and thus elicit little or no antiviral inflammatory response [12,32]. Because RSV infection within the earliest months of life is most clearly related to subsequent development of asthma, and because previous studies have established that the pattern of host response to pneumovirus infection is age-dependent [33,34], we specifically examined responses only in mice infected with PVM in the first two days of life.

To further emulate the earliest phases of induction of asthma in childhood, our model involved sensitisation via the respiratory tract. We then employed chronic low-level challenge with aerosolised OVA followed by a single moderate-level challenge, using mass concentrations of aerosolised antigen at least 10-100 fold lower than in commonly used short-term experimental systems [18]. The magnitude of the inflammatory response observed was therefore relatively modest, but as in our previously described models based on systemic sensitisation [17,18], we elicited key features of chronic asthma that resembled the human disease and were limited to conducting airways (eosinophil recruitment into the epithelial layer, chronic inflammation in the airway wall, and changes of remodelling such as subepithelial fibrosis, epithelial hypertrophy and goblet cell metaplasia). Following the final moderate-level challenge, we also elicited features of an exacerbation of asthma (distal airway inflammation with increased recruitment of eosinophils).

Our key finding was that it was only in the setting of early-life viral infection followed by sensitisation via the respiratory tract that several crucial elements of the asthmatic phenotype developed, with allergen challenge then able to elicit inflammation typical of both chronic stable asthma and an acute exacerbation. Recovery from viral infection, or allergen sensitisation and challenge, were separately each able to induce some elements of airway inflammation, remodelling or hyperresponsiveness to a cholinergic stimulus. The pattern of responses is summarised in Table 5 and provides insight into the relative contributions of prior viral infection and allergen exposure to the development of the lesions of childhood asthma.

**Table 5:** Summary of responses of key experimental groups

	Allergic inflammation		Th2 response		Airway remodelling		AHR
Early-life infection alone	-		-		+		+++
Sensitisation and challenge alone	+		±		+++		+
Infection followed by sensitisation and challenge	+++		+++		+++		+++
Infection followed by sensitisation and challenge in IL-4Rα^-/- ^mice	-		-		++		-

Specifically, the co-operative interaction between early-life infection and allergen challenge for induction of asthmatic inflammation was apparent in terms of recruitment of eosinophils into the airways, as well as corresponding increases in the number of BAL cells and of CD4^+ ^T cells recovered from lung tissue. Inflammation was also closely allied with development of a Th2-biased immunological response. Mucous cell change and AHR were associated with recovery from early-life viral infection alone, but like asthmatic inflammation, were dependent on signalling via the IL-4 receptor. In contrast, we found that changes of subepithelial fibrosis and epithelial hypertrophy in the conducting airways were associated with allergen sensitisation and chronic challenge alone. Airway remodelling did not appear to be dependent on either signalling via the IL-4 receptor or a Th2-biased response, but could be related to production of growth factors such as TGF-β and IGF-1 by airway epithelial cells, which we have previously demonstrated following chronic inhalational challenge [35].

Our data are consistent with findings in biopsy studies of children either at risk for or with established asthma, which suggest that subepithelial fibrosis develops during the early induction phase. Indeed there is now considerable evidence that, contrary to previously held views, changes of remodelling precede or occur in parallel with inflammation, and are evident well before the diagnosis of childhood asthma [36-40]. They are also consistent with patient data indicating that AHR may be dissociated from asthmatic inflammation and precede the development of clinical asthma [36,41]. However, the relationship between subepithelial fibrosis, airway inflammation and AHR has not previously been assessed in an experimental model of childhood asthma.

In the present study, AHR was primarily related to recovery from neonatal infection with PVM, as well as to expression of mRNA for *Gob5 *and to goblet cell hyperplasia/metaplasia. *Gob5 *(also known as *mClca3*) is a calcium-dependent chloride channel which has key roles in the development of mucous cell change and AHR [42,43], so this correlation is of particular interest. Enhanced expression of *Gob5 *is likely to be a response to injury of epithelial cells, which are the primary site of replication of pneumoviruses. As noted above, AHR was also dependent on signalling via the IL-4 receptor, suggesting a role for cytokines such as IL-4 and IL-13. Previous studies of RSV infection in weanling mice have demonstrated induction of AHR that may persist for up to 24 weeks [44], which is consistent with the presence of AHR in mice which recovered from neonatal infection with PVM.

Our study also provides evidence that in this model, the underlying mechanism of development of an asthmatic inflammatory response is recruitment and activation of pulmonary T cells, with induction of a Th2-biased immunological response. Only in animals that recovered from a neonatal infection with PVM, then were sensitised by intranasal administration of OVA and subsequently challenged with aerosolised allergen was there evidence of high levels of anti-OVA IgE and IgG_1_, together with increased numbers of pulmonary CD4^+ ^cells which expressed elevated levels of mRNA for IL-4, IL-5, IL-13 and GATA-3, as well as enhanced secretion of IL-4, IL-5 and IL-13. In IL-4Rα^-/- ^mice, in which Th2 responses are abrogated because the IL-4 receptor α chain is involved in signalling for both IL-4 and IL-13, development of asthmatic inflammation, mucous cell change or AHR was prevented. Evidence of the importance of induction of a Th2-biased response is of particular interest, as this is clearly of clinical relevance in childhood asthma [45] and recent studies suggest that inhibition of IL-4Rα can reduce some of the clinical manifestations in patients with atopic asthma [46]. Furthermore, our observations suggest a key role for early-life viral infection in breaking tolerance to antigen, because neonatal exposure to ovalbumin via the respiratory tract otherwise induces tolerance [47,48]. However, the timing of sensitisation had a critical impact on the development of asthmatic lesions, so that inflammation and changes of remodelling were either reduced or absent in animals sensitised on days 1 and 2 of life.

A possible mechanism for induction of Th2 responses is revealed by our finding that IL-25 was upregulated in lung tissue of 14-28 day old animals following neonatal PVM infection. IL-25 is expressed by airway epithelial cells and has been implicated in upregulating GATA-3 expression and therefore promoting Th2 differentiation [49]. Furthermore, IL-25 co-operates with other cytokines in activating dendritic cells to upregulate expression of class II MHC and various costimulatory molecules, which collectively function as instructive signals that drive Th2 polarisation [50,51]. Therefore, recruitment and activation of dendritic cells will be a focus of future investigations using this model. IL-25 may also have direct effects on development of an asthmatic response: when delivered via the airways, this cytokine induces inflammation, production of Th2 cytokines and AHR which appears to be dependent on induction of IL-13 via a STAT6-mediated pathway [52].

Our observations in this model provide direct experimental support for a number of epidemological studies. We recognise that genetic factors may play crucial roles in determining susceptibility to development of allergic asthma following early-life viral infection [7] and it is possible that our experimental data are significantly influenced by use of the BALB/c strain of mice, which is predisposed to the development of Th2-biased immune responses. Nevertheless, this model provides hitherto unavailable opportunities for investigation of the molecular mechanisms underlying progression of asthma in childhood.

## Conclusion

We have demonstrated that in a novel clinically relevant experimental model, the interplay between an early-life pneumovirus infection and subsequent ongoing allergen challenge is crucial to induction of key features of childhood asthma. Elements of the asthmatic phenotype could be induced following either recovery from viral infection or allergen sensitisation and challenge. However, only in the setting of early-life viral infection followed by sensitisation via the respiratory tract and inhalational challenge did animals develop the full constellation of features, including acute and chronic inflammation of the airways accompanied by a Th2-biased immunological response which contributed to their development.

## List of Abbreviations

AHR: airway hyperresponsiveness; BAL: bronchoalveolar lavage; ELISpot: enzyme-linked immunospot; HRP: horseradish peroxidase; Ig: immunoglobulin; IL: interleukin; IFN: interferon; OCT: optimal cutting temperature; OVA: ovalbumin; PAS: periodic acid schiff; PBS: phosphate buffered saline; PFU: plaque forming units; PVM: pneumonia virus of mice; R_L_: transpulmonary resistance; RNA: ribonucleic acid; RSV: respiratory syncytial virus; RT-PCR: real-time polymerase chain reaction; STAT: signal transduction and activator of transcription; Th: T-helper cell.

## Competing interests

The authors declare that they have no competing interests.

## Authors' contributions

JSS contributed to the design of the study, was primarily responsible for the experimental studies and analysis of the data, and drafted the initial version of the manuscript. NH performed the early-life infection/sensitisation and was responsible for various other aspects of the experimental work. CH assisted with aspects of the experimental work and contributed to editing of the manuscript. HFR and JBD developed the early-life infection model and contributed to editing of the manuscript. KLA assisted with aspects of the experimental work. PSF and RKK conceived the study, participated in its design and co-ordination, and helped to draft the final version of the manuscript. All authors read and approved the final manuscript.

## References

[B1] Wong GW Chow CM Childhood asthma epidemiology: insights from comparative studies of rural and urban populations Pediatr Pulmonol 2008 43 2 107 116 10.1002/ppul.20755 18092349

[B2] Garn H Renz H Epidemiological and immunological evidence for the hygiene hypothesis Immunobiology 2007 212 441 452 10.1016/j.imbio.2007.03.006 17544829

[B3] Holt PG Sly PD Interactions between RSV infection, asthma, and atopy: unraveling the complexities J Exp Med 2002 196 10 1271 1275 10.1084/jem.20021572 12438419 PMC2193993

[B4] Holt PG Sly PD Prevention of allergic respiratory disease in infants: current aspects and future perspectives Curr Opin Allergy Clin Immunol 2007 7 6 547 555 10.1097/ACI.0b013e3282f14a17 17989533

[B5] Jackson DJ Gangnon RE Evans MD Roberg KA Anderson EL Pappas TE Printz MC Lee WM Shult PA Reisdorf E Wheezing rhinovirus illnesses in early life predict asthma development in high-risk children Am J Respir Crit Care Med 2008 178 7 667 672 10.1164/rccm.200802-309OC 18565953 PMC2556448

[B6] Kusel MM de Klerk NH Kebadze T Vohma V Holt PG Johnston SL Sly PD Early-life respiratory viral infections, atopic sensitization, and risk of subsequent development of persistent asthma J Allergy Clin Immunol 2007 119 5 1105 1110 10.1016/j.jaci.2006.12.669 17353039 PMC7125611

[B7] Lemanske RF Viral infections and asthma inception J Allergy Clin Immunol 2004 114 1023 1026 10.1016/j.jaci.2004.08.031 15536404

[B8] Sigurs N Gustafsson PM Bjarnason R Lundberg F Schmidt S Sigurbergsson F Kjellman B Severe respiratory syncytial virus bronchiolitis in infancy and asthma and allergy at age 13 Am J Respir Crit Care Med 2005 171 137 141 10.1164/rccm.200406-730OC 15516534

[B9] Kristjansson S Bjarnarson SP Wennergren G Palsdottir AH Arnadottir T Haraldsson A Jonsdottir I Respiratory syncytial virus and other respiratory viruses during the first 3 months of life promote a local TH2-like response J Allergy Clin Immunol 2005 116 4 805 811 10.1016/j.jaci.2005.07.012 16210054

[B10] Simoes EA Respiratory syncytial virus infection Lancet 1999 354 9181 847 852 10.1016/S0140-6736(99)80040-3 10485741

[B11] Domachowske JB Bonville CA Rosenberg HF Animal models for studying respiratory syncytial virus infection and its long term effects on lung function Pediatr Infect Dis J 2004 23 S228 S234 10.1097/01.inf.0000144672.81955.a4 15577578

[B12] Mohapatra SS Boyapalle S Epidemiologic, experimental, and clinical links between respiratory syncytial virus infection and asthma Clin Microbiol Rev 2008 21 3 495 504 10.1128/CMR.00054-07 18625684 PMC2493089

[B13] Rosenberg HF Bonville CA Easton AJ Domachowske JB The pneumonia virus of mice infection model for severe respiratory syncytial virus infection: identifying novel targets for therapeutic intervention Pharmacol Ther 2005 105 1 1 6 10.1016/j.pharmthera.2004.09.001 15626452

[B14] Rosenberg HF Domachowske JB Pneumonia virus of mice: severe respiratory infection in a natural host Immunol Lett 2008 118 1 6 12 10.1016/j.imlet.2008.03.013 18471897 PMC2494858

[B15] Bonville CA Bennett NJ Koehnlein M Haines DM Ellis JA DelVecchio AM Rosenberg HF Domachowske JB Respiratory dysfunction and proinflammatory chemokines in the pneumonia virus of mice (PVM) model of viral bronchiolitis Virology 2006 349 1 87 95 10.1016/j.virol.2006.02.017 16563455

[B16] Bonville CA Percopo CM Dyer KD Gao J Prussin C Foster B Rosenberg HF Domachowske JB Interferon-gamma coordinates CCL3-mediated neutrophil recruitment in vivo BMC Immunol 2009 10 14 10.1186/1471-2172-10-14 19298652 PMC2662797

[B17] Temelkovski J Hogan SP Shepherd DP Foster PS Kumar RK An improved murine model of asthma: selective airway inflammation, epithelial lesions and increased methacholine responsiveness following chronic exposure to aerosolised allergen Thorax 1998 53 849 856 10.1136/thx.53.10.849 10193371 PMC1745083

[B18] Siegle JS Hansbro N Herbert C Yang M Foster PS Kumar RK Airway hyperreactivity in exacerbation of chronic asthma is independent of eosinophilic inflammation Am J Respir Cell Mol Biol 2006 35 565 570 10.1165/rcmb.2006-0135OC 16794258

[B19] Noben-Trauth N Shultz LD Brombacher F Urban JF Jr Gu H Paul WE An interleukin 4 (IL-4)-independent pathway for CD4+ T cell IL-4 production is revealed in IL-4 receptor-deficient mice Proc Natl Acad Sci USA 1997 94 20 10838 10843 10.1073/pnas.94.20.10838 9380721 PMC23501

[B20] Weil SC Hrisinko MA A hybrid eosinophilic-basophilic granulocyte in chronic granulocytic leukemia Am J Clin Pathol 1987 87 1 66 70 2432775 10.1093/ajcp/87.1.66

[B21] Zucker-Franklin D Grusky G The identification of eosinophil colonies in soft-agar cultures by differential staining for peroxidase J Histochem Cytochem 1976 24 12 1270 1272 63511 10.1177/24.12.63511

[B22] Ameredes BT Cardiac activity during airway resistance alterations with intravenous and inhaled methacholine Respir Physiol Neurobiol 2004 139 281 292 10.1016/j.resp.2003.10.010 15122994

[B23] Asquith KL Ramshaw HS Hansbro PM Beagley KW Lopez AF Foster PS The IL-3/IL-5/GM-CSF common receptor plays a pivotal role in the regulation of Th2 immunity and allergic airway inflammation J Immunol 2008 180 2 1199 1206 18178860 10.4049/jimmunol.180.2.1199

[B24] Ellis JA Martin BV Waldner C Dyer KD Domachowske JB Rosenberg HF Mucosal inoculation with an attenuated mouse pneumovirus strain protects against virulent challenge in wild type and interferon-gamma receptor deficient mice Vaccine 2007 25 6 1085 1095 10.1016/j.vaccine.2006.09.081 17052820 PMC1922442

[B25] Garvey TL Dyer KD Ellis JA Bonville CA Foster B Prussin C Easton AJ Domachowske JB Rosenberg HF Inflammatory responses to pneumovirus infection in IFN-alpha beta R gene-deleted mice J Immunol 2005 175 7 4735 4744 16177121 10.4049/jimmunol.175.7.4735

[B26] Yi X Feng F Xiang Z Ge L The effects of allitridin on the expression of transcription factors T-bet and GATA-3 in mice infected by murine cytomegalovirus J Med Food 2005 8 3 332 336 10.1089/jmf.2005.8.332 16176143

[B27] Moyron-Quiroz JE Rangel-Moreno J Kusser K Hartson L Sprague F Goodrich S Woodland DL Lund FE Randall TD Role of inducible bronchus associated lymphoid tissue (iBALT) in respiratory immunity Nat Med 2004 10 9 927 934 10.1038/nm1091 15311275

[B28] Purkerson J Isakson P A two-signal model for regulation of immunoglobulin isotype switching FASEB J 1992 6 14 3245 3252 1385241 10.1096/fasebj.6.14.1385241

[B29] Hansbro NG Horvat JC Wark PA Hansbro PM Understanding the mechanisms of viral induced asthma: New therapeutic directions Pharmacol Ther 2008 117 3 313 353 10.1016/j.pharmthera.2007.11.002 18234348 PMC7112677

[B30] Rosenberg HF Domachowske JB Eosinophils, eosinophil ribonucleases, and their role in host defense against respiratory virus pathogens 2001 70 691 698 11698487

[B31] Barends M de Rond LG Dormans J van Oosten M Boelen A Neijens HJ Osterhaus AD Kimman TG Respiratory syncytial virus, pneumonia virus of mice, and influenza A virus differently affect respiratory allergy in mice Clin Exp Allergy 2004 34 488 496 10.1111/j.1365-2222.2004.01906.x 15005745

[B32] Holtzman MJ Tyner JW Kim EY Lo MS Patel AC Shornick LP Agapov E Zhang Y Acute and chronic airway responses to viral infection: implications for asthma and chronic obstructive pulmonary disease Proc Am Thorac Soc 2005 2 2 132 140 10.1513/pats.200502-015AW 16113481 PMC2713316

[B33] Culley FJ Pollott J Openshaw PJ Age at first viral infection determines the pattern of T cell-mediated disease during reinfection in adulthood J Exp Med 2002 196 10 1381 1386 10.1084/jem.20020943 12438429 PMC2193991

[B34] Dakhama A Park JW Taube C Joetham A Balhorn A Miyahara N Takeda K Gelfand EW The enhancement or prevention of airway hyperresponsiveness during reinfection with respiratory syncytial virus is critically dependent on the age at first infection and IL-13 production J Immunol 2005 175 1876 1883 16034131 10.4049/jimmunol.175.3.1876

[B35] Herbert C Hettiaratchi A Webb DC Thomas PS Foster PS Kumar RK Suppression of cytokine expression by roflumilast and dexamethasone in a model of chronic asthma Clin Exp Allergy 2008 38 5 847 856 10.1111/j.1365-2222.2008.02950.x 18307529

[B36] Laprise C Laviolette M Boutet M Boulet LP Asymptomatic airway hyperresponsiveness: relationships with airway inflammation and remodelling Eur RespirJ 1999 14 63 73 10.1034/j.1399-3003.1999.14a12.x 10489830

[B37] Fedorov IA Wilson SJ Davies DE Holgate ST Epithelial stress and structural remodelling in childhood asthma Thorax 2005 60 5 389 394 10.1136/thx.2004.030262 15860714 PMC1758889

[B38] Pohunek P Warner JO Turzikova J Kudrmann J Roche WR Markers of eosinophilic inflammation and tissue re-modelling in children before clinically diagnosed bronchial asthma Pediatr Allergy Immunol 2005 16 1 43 51 10.1111/j.1399-3038.2005.00239.x 15693911

[B39] Barbato A Turato G Baraldo S Bazzan E Calabrese F Panizzolo C Zanin ME Zuin R Maestrelli P Fabbri LM Epithelial damage and angiogenesis in the airways of children with asthma Am J Respir Crit Care Med 2006 174 975 981 10.1164/rccm.200602-189OC 16917118

[B40] Saglani S Payne DN Zhu J Wang Z Nicholson AG Bush A Jeffery PK Early detection of airway wall remodeling and eosinophilic inflammation in preschool wheezers Am J Respir Crit Care Med 2007 176 9 858 864 10.1164/rccm.200702-212OC 17702968

[B41] Hopp RJ Townley RG Biven RE Bewtra AK Nair NM The presence of airway reactivity before the development of asthma Am Rev Respir Dis 1990 141 1 2 8 2404438 10.1164/ajrccm/141.1.2

[B42] Nakanishi A Morita S Iwashita H Sagiya Y Ashida Y Shirafuji H Fujisawa Y Nishimura O Fujino M Role of gob-5 in mucus overproduction and airway hyperresponsiveness in asthma Proc Natl Acad Sci USA 2001 98 5175 5180 10.1073/pnas.081510898 11296262 PMC33183

[B43] Long AJ Sypek JP Askew R Fish SC Mason LE Williams CM Goldman SJ Gob-5 contributes to goblet cell hyperplasia and modulates pulmonary tissue inflammation Am J Respir Cell Mol Biol 2006 35 357 365 10.1165/rcmb.2005-0451OC 16645179

[B44] Collins RA Gualano RC Zosky GR Chiappetta CL Turner DJ Colasurdo GN Hantos Z Sly PD Lack of long-term effects of respiratory syncytial virus infection on airway function in mice Respir Physiol Neurobiol 2007 156 345 352 10.1016/j.resp.2006.11.009 17236822

[B45] Anderson GP Endotyping asthma: new insights into key pathogenic mechanisms in a complex, heterogeneous disease Lancet 2008 372 9643 1107 1119 10.1016/S0140-6736(08)61452-X 18805339

[B46] Wenzel S Wilbraham D Fuller R Getz EB Longphre M Effect of an interleukin-4 variant on late phase asthmatic response to allergen challenge in asthmatic patients: results of two phase 2a studies Lancet 2007 370 9596 1422 1431 10.1016/S0140-6736(07)61600-6 17950857

[B47] Gerhold K Bluemchen K Franke A Stock P Hamelmann E Exposure to endotoxin and allergen in early life and its effect on allergen sensitization in mice J Allergy Clin Immunol 2003 112 389 396 10.1067/mai.2003.1646 12897747

[B48] Wang Y McCusker C Neonatal exposure with LPS and/or allergen prevents experimental allergic airways disease: development of tolerance using environmental antigens J Allergy Clin Immunol 2006 118 143 151 10.1016/j.jaci.2006.03.020 16815150

[B49] Angkasekwinai P Park H Wang YH Wang YH Chang SH Corry DB Liu YJ Zhu Z Dong C Interleukin 25 promotes the initiation of proallergic type 2 responses J Exp Med 2007 204 1509 1517 10.1084/jem.20061675 17562814 PMC2118650

[B50] Lloyd CM Dust mites' dirty dealings in the lung Nat Med 2009 15 366 367 10.1038/nm0409-366 19350005 PMC3381716

[B51] Lambrecht BN Hammad H Biology of lung dendritic cells at the origin of asthma Immunity 2009 31 412 424 10.1016/j.immuni.2009.08.008 19766084

[B52] Sharkhuu T Matthaei KI Forbes E Mahalingam S Hogan SP Hansbro PM Foster PS Mechanism of interleukin-25 (IL-17E)-induced pulmonary inflammation and airways hyper-reactivity Clin Exp Allergy 2006 36 1575 1583 10.1111/j.1365-2222.2006.02595.x 17177681

